# Diagnostic Delays in Pediatric Acute Ischemic Stroke: 24-Year Trends and Contributing Factors in Switzerland

**DOI:** 10.1161/STROKEAHA.125.054402

**Published:** 2026-05-01

**Authors:** Daniel Brechbühl, Regula Everts, Barbara Goeggel-Simonetti, Esmeralda Nava, Catherine Wohlgemuth, Florian Bauder, Gabriela Oesch, Joel Fluss, Madeleine Bachmann, Nicole Faignart, Susi Strozzi, Robin Muenger, Stephane Darteyre, Maja Steinlin

**Affiliations:** Division of Neuropaediatrics, Development and Rehabilitation, Department of Paediatrics, Inselspital, Bern University Hospital, University of Bern, Switzerland (D.B., R.E., G.O., R.M., M.S.).; Graduate School for Health Sciences, University of Bern, Switzerland (D.B., R.M.).; Department of Neuropediatrics, Institute of Pediatrics of Southern Switzerland, San Giovanni Hospital, Bellinzona, Switzerland (B.G.-S.).; Faculty of Biomedical Sciences, Università della Svizzera Italiana, Lugano, Switzerland (B.G.-S.).; Department of Pediatric Neurology, Cantonal Hospital Aarau, Switzerland (E.N.).; Department of Pediatric Neurology/Epileptology, University Children’s Hospital Zurich, Switzerland (C.W.).; Social Paediatrics Center, Cantonal Hospital Winterthur, Switzerland (F.B.).; Pediatric Neurology Unit, Geneva Children’s Hospital, Switzerland (J.F.).; Department of Child Neurology, Children’s Hospital of Eastern Switzerland, St. Gallen (M.B.).; Department of Pediatrics, Hôpital du Valais, Sion, Switzerland (N.F.).; Department of Pediatrics, Kantonsspital Graubünden, Chur, Switzerland (S.S.).; University Children’s Hospital Basel, Switzerland (R.M.).; Pediatric Neurology and Neurorehabilitation Unit, Lausanne University Hospital, Switzerland (S.D.).

**Keywords:** child, delayed diagnosis, ischemic stroke, reperfusion, thrombectomy, thrombolytic therapy

## Abstract

**BACKGROUND:**

Growing evidence for reperfusion therapies in pediatric acute ischemic stroke (AIS) increases the importance of timely diagnosis within treatment windows. We therefore aimed to describe 24-year trends and associated factors of diagnostic delay.

**METHODS:**

We conducted a nationwide retrospective cross-sectional study including 314 children aged 28 days to 16 years from the Swiss Neuropediatric Stroke Registry (2000–2023). The primary outcome was time from stroke onset to diagnosis (TOD). Trends for diagnoses beyond intravenous thrombolysis (≥4.5 hours) and thrombectomy (≥24 hours) windows were assessed with multivariable logistic regression, and for continuous TOD with robust linear regression. Prespecified covariates were retained in multivariable models if associated with the outcome in univariable analyses. Analyses were stratified by AIS onset location (out-of-hospital and in-hospital).

**RESULTS:**

Median TOD was 26.9 hours (interquartile range, 10.1–91.5) between 2000 and 2023. During this period, the proportion diagnosed beyond the thrombolysis window decreased significantly in the overall cohort from 90.9% to 77.5% (adjusted odds ratio per calendar year 0.94 [95% CI, 0.88–1.00]) and in out-of-hospital AIS from 88.1% to 74.1% (adjusted odds ratio, 0.91 [0.84–1.00]). No significant change was observed beyond the thrombectomy window diagnoses. Continuous TOD decreased significantly only in in-hospital AIS (β=−4.3 [−7.2 to −1.5]). Older age (β=−1.8 [−2.9 to −0.8]), higher pediatric National Institutes of Health Stroke Scale (β=−1.2 [−2.1 to −0.4]), and facial palsy (β=−19.4 [−30.2 to −8.5]) were associated with shorter TOD, and nonspecific symptoms (β=110.0 [72.5–147.5]) with longer TOD. Out-of-hospital TOD was shorter when patients presented to a stroke center compared with other presentation sites. Posterior-stroke symptoms were associated with diagnoses beyond the thrombolysis window.

**CONCLUSIONS:**

Despite decreasing proportions of beyond-thrombolysis window diagnoses in out-of-hospital AIS and decreasing TOD in in-hospital AIS, most diagnoses occur beyond reperfusion windows. Goals to decrease delay include raising awareness of posterior-stroke signs and AIS in younger children, and strengthening direct-to-stroke center pathways.

With growing evidence supporting reperfusion therapies^1,2^ for the treatment of pediatric acute ischemic stroke (AIS), timely diagnosis is becoming increasingly critical for the following reasons: Access for potential candidates and the efficacy of reperfusion are largely limited to the 2 temporal windows of 4.5 hours for intravenous thrombolysis and 24 hours for endovascular thrombectomy.^3,4^ Earlier reperfusion is consistently associated with better outcomes, while experimental data show that infarct volume enlarges with ongoing arterial occlusion.^2,5^ Despite this urgency, the median time from stroke onset to diagnosis (TOD) in pediatric patients is reported to range between 22.7 to 27 hours.^6–9^ By contrast, diagnostic delays in adults in Switzerland are shorter, typically within a few hours from stroke onset.^10^ Limited awareness of pediatric stroke and the challenge of recognizing stroke-specific symptoms contribute substantially to diagnostic delays.^7,11^ Additional barriers include the limited availability of stroke protocols and specialized infrastructure in pediatric centers.^12^ The higher prevalence of stroke mimics in children further complicates triage and decision-making in emergency settings.^13^ To improve early recognition, especially by laypersons and first responders, the Face-Arm-Speech-Time test (FAST) was introduced around the turn of the millennium.^14^ It was later expanded to Balance-Eyes-Face-Arm-Speech-Time test (BEFAST), adding “Balance” and “Eyes” to improve detection of posterior circulation strokes.^15^ Public awareness campaigns using FAST have led to reduced diagnostic delays in adult patients with stroke.^16^ In emergency settings, BEFAST implementation has also been associated with reduced delay in time from admission to diagnosis.^17^ In parallel, the integration of stroke management protocols into pediatric healthcare has increased in recent years.^18^ Their implementation has been associated with a reduction in admission-to-diagnosis time.^19^ In Canada a notable reduction in onset-to-admission time has been observed, as demonstrated by findings from a recent 16-year study in a pediatric stroke cohort.^20^ However, it remains unclear whether similar improvements have occurred in other countries and whether increased awareness has translated into a higher proportion of children being diagnosed within therapeutic time windows.

The primary aim of this study was to assess whether the TOD in children with AIS has decreased over the past 24 years in Switzerland. Secondary objectives were to evaluate whether the number of children diagnosed within the thrombolysis and thrombectomy time windows has increased, to analyze changes in onset-to-admission and admission-to-diagnosis time, and to identify patient-related, system-level, and clinical factors associated with diagnostic delays. We hypothesized that TOD has decreased over time and is shorter in children with in-hospital versus out-of-hospital stroke onset and in children with FAST-positive symptoms versus isolated balance or eye symptoms (only B&E symptoms).

## Methods

The reporting of this retrospective cross-sectional study of an existing clinical registry database follows the STROBE guidelines (Strengthening the Reporting of Observational Studies in Epidemiology) for observational research (Supplemental Material).^21^ The data that support the findings of this study are available from the corresponding author upon reasonable request, subject to applicable restrictions.

### Patient Population

The Swiss Neuropediatric Stroke Registry (SNPSR) is a nationwide, prospective, population-based registry for pediatric stroke. The registry was screened for children who experienced AIS between January 1, 2000, and December 31, 2023. Cases were eligible for inclusion if they had a confirmed diagnosis of arterial ischemic stroke, defined by stroke-specific clinical symptoms and corresponding findings on neuroimaging, were between 28 days and 16 years of age, and experienced the stroke within Switzerland. The upper age limit of 16 years was chosen because, in the Swiss healthcare system, adolescents aged ≥16 years are routinely managed within adult neurology services. Cases were excluded if the categorical variable for TOD was missing.

### Definitions

In-hospital stroke was defined as a stroke occurring in patients admitted for reasons other than stroke. Out-of-hospital stroke was defined as a stroke with stroke onset before hospital admission. Stroke onset was used as an umbrella term, defined as the earliest reported neurological abnormality when onset was witnessed and as the last known well when onset was unwitnessed. If only nonspecific symptoms (eg, personality changes or headaches) were reported, stroke onset was marked at the start of those symptoms. For cases where stroke onset was documented in general terms (eg, morning) or an exact timepoint was missing, the earliest plausible time within the specified period was used as an approximation. Admission time was recorded as the initial healthcare contact. Diagnosis time corresponded to the first imaging sequence confirming the diagnosis, unless otherwise specified in the SNPSR. To minimize observer bias, all timepoints were evaluated by the same assessor using standardized definitions for the timepoints, as outlined above. In instances where the SNPSR data set contained incomplete information, the respective hospitals were contacted to obtain the missing data.

In this study, pediatric stroke centers were defined as those pediatric hospitals that are affiliated with a Swiss Federation of Clinical Neuro-Societies (SFCNS; https://sfcns.ch/certification/stroke) certified adult stroke center.^22^

### Time Interval Calculation

Time intervals, including TOD, onset-to-admission, and admission-to-diagnosis time, were prerecorded for some cases in the SNPSR. For others, these intervals were calculated as continuous variables based on the defined timepoints. Continuous time intervals are reported as medians with interquartile ranges (IQRs). All intervals were subsequently stratified into predefined categorical groups for analysis. TOD was categorized using a cutoff of 4.5 hours and 24 hours, reflecting the upper limit of the intravenous thrombolysis and of the thrombectomy window, respectively. The onset-to-admission interval was categorized using <3 hours, 3 to 24 hours, and >24 hours, reflecting the assumption that most children would present within the first day. For the admission-to-diagnosis interval, we applied <1 hour, 1 to 3 hours, and >3 hours, based on the clinical benchmark that door-to-needle time should not exceed 1 hour.^23^ When continuous variables could not be determined due to missing or incomplete timepoint data, classification into categorical groups was still performed if sufficient information was available to allow for reliable categorization.

### Demographic and Clinical Data

Baseline characteristics were extracted from the SNPSR and included demographics, presenting symptoms, and the type of first point of contact facility (outpatient provider, nonstroke center, pediatric stroke center). For the regression models, the numerical coding of the presentation-site variable (0=outpatient provider, 1=nonstroke center, 2=pediatric stroke center) was chosen to reflect expected system-related delays. A separate comparison of TOD across these categories was performed to confirm that this coding order corresponded to the observed delay patterns.

For the delay in onset-to-diagnosis time associated with symptom analysis, symptoms reported by caregivers or patients were used. For all other intervals, symptoms were considered present if reported by caregivers or observed during clinical examination. In evaluating the BEFAST criteria, vertigo was included under Balance, but a decreased level of consciousness alone was not sufficient for BEFAST fulfillment. Nonspecific symptoms were defined as the absence of BEFAST symptoms, epileptic seizures, or decreased level of consciousness. For analyses of the onset-to-admission interval, children were classified as B&E-positive if caregivers reported only B or E symptoms. For all other intervals, children were considered B&E-positive only if neither caregivers nor clinicians observed any FAST-positive symptoms.

Socioeconomic status was estimated using the Swiss Neighborhood Index of Socioeconomic Position (SSEP),^24^ with SSEP-1 applied to strokes before 2012 and SSEP-3 thereafter. Patient addresses and pediatric stroke center locations were used to calculate distances via Google Maps.

### Post Hoc Analyses

As a post hoc analysis, TOD among in-hospital AIS cases was compared between groups stratified by postoperative sedated state. The postoperative sedated state was defined as a surgical procedure followed by a reduced level of consciousness.

### Ethical Aspects

The SNPSR operates under ethical approval of the cantonal ethics committee of Bern (BASEC-ID: 2025-00217). Comprehensive details about the SNPSR are available in prior publications.^25^ Written informed consent from parents or legal guardians is collected upon registry enrollment.

### Missing Data

Patients with incomplete data for specific intervals were excluded from the analysis of those intervals.

### Statistics

Descriptive statistics were used to summarize patient characteristics across study periods. Normality of continuous variables was evaluated visually using histograms and Q-Q plots and analytically using the Shapiro-Wilk test and skewness measures. Corresponding plots and test results are provided in the Supplemental Material (Table S1; Figures S1 through S5). Results indicated non-normal distributions. When comparing small cell sizes (n<10), Fisher exact test was used to compare categorical variables, and the Wilcoxon rank-sum test was used to compare continuous variables. For Wilcoxon tests, effect sizes were additionally reported as Rosenthal’s r (Z/√N). Continuous time intervals were analyzed using robust linear regression to account for outliers, while categorical time intervals were assessed using logistic regression. Robust linear regression was chosen because it reduces the influence of extreme values and violations of normality assumptions compared with ordinary least squares regression.^26^ Potential confounders were predefined based on clinical and conceptual relevance and availability. These included patient-related factors (age, socioeconomic status, year of stroke, weekend onset, wake-up stroke), system- and access-related factors (distance to the nearest stroke center, interhospital transfer, first imaging modality, and location of first presentation), and clinical characteristics (pediatric National Institutes of Health Stroke Scale, hemiparesis, headache, vertigo, visual disturbances, speech deficits, facial palsy, balance problems, decreased level of consciousness, seizures, and patterns limited to nonspecific or isolated B&E symptoms of the BEFAST). For each multivariable time-interval model, appropriate confounders were identified using a univariable screening approach with a significance threshold of *P*<0.05. Because multivariable models analyzed prespecified associations within a single regression framework rather than multiple independent tests, no formal multiple-comparison adjustment was applied. In contrast, Holm-Bonferroni correction was used for exploratory nonparametric pairwise comparisons.

A 2-sided *P*<0.05 was considered statistically significant. All analyses were performed using Stata, version 16.1 (StataCorp), and data visualizations were created in R, version 4.4.1.

## Results

### Participants

**Figure 1.**
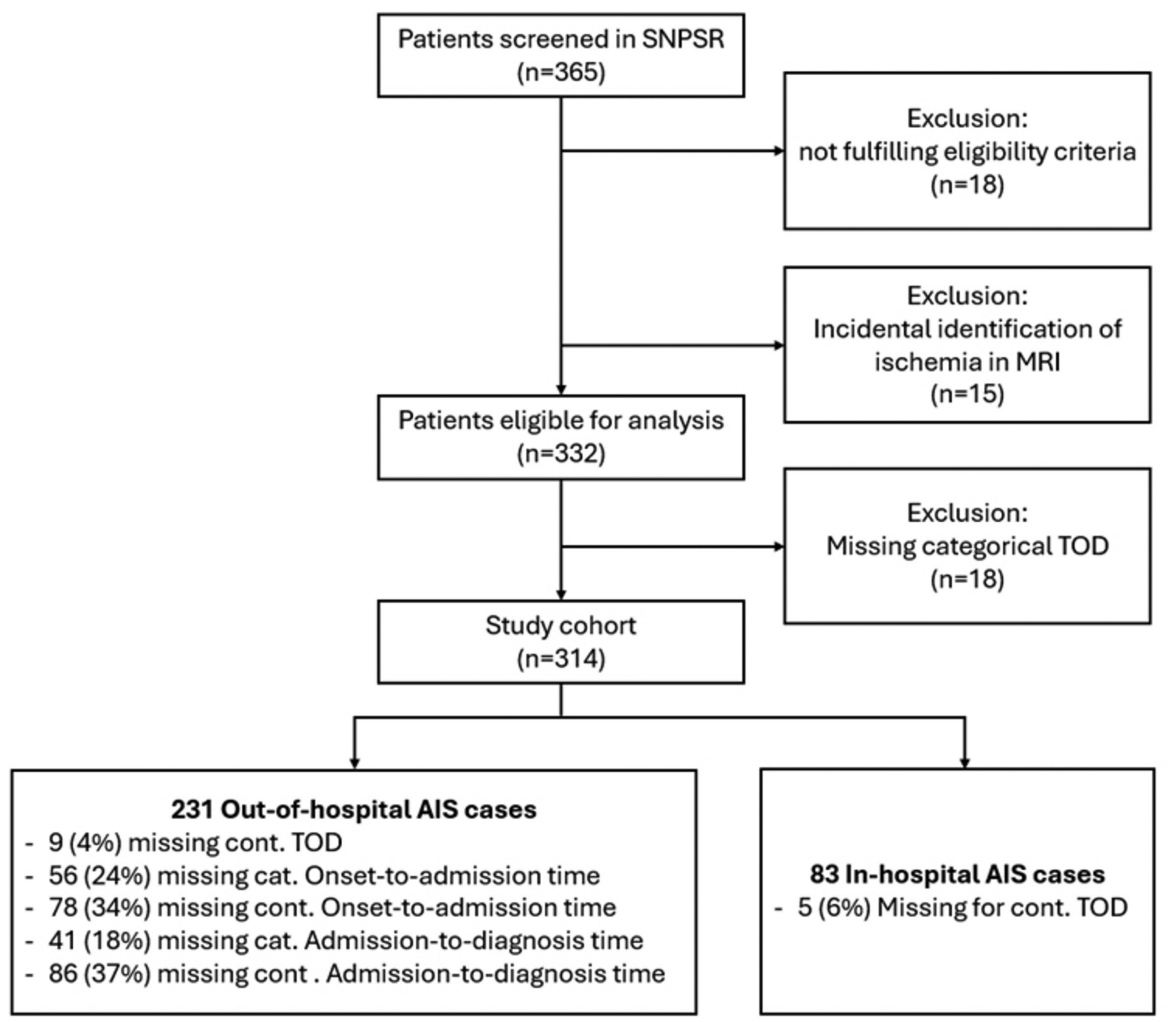
**Flowchart illustrating the selection of the study population and missing values for each category.** AIS indicates acute ischemic stroke; cat., categorical variables; cont., continuous variables; MRI, magnetic resonance imaging; SNPSR, Swiss Neuropediatric Stroke Registry; and TOD, time from stroke onset to diagnosis.

A total of 365 children were screened, of whom 332 met the eligibility criteria for inclusion in the study. Of these, 314 cases (231 out-of-hospital AIS cases and 83 in-hospital cases) were categorically assigned to a TOD group. For 300 cases, TOD was determined as a continuous variable. Patients with incomplete data for specific time intervals were excluded from the analysis of those intervals. Among out-of-hospital AIS cases (n=231), continuous TOD was missing in 94 (41%), onset-to-admission time in 56 (24%), and admission-to-diagnosis time in 41 (18%) cases. Among in-hospital AIS cases (n=83), continuous TOD was unavailable in 5 (6%) cases (see Figure 1).

The baseline characteristics are summarized in Table 1. The male-to-female ratio across the study population was 3:2. Median age was higher in the out-of-hospital AIS group (6.6 years [IQR, 2.7–11.8]) compared with the in-hospital AIS group (2.3 years [IQR, 0.4–8.8]).

**Table 1.**
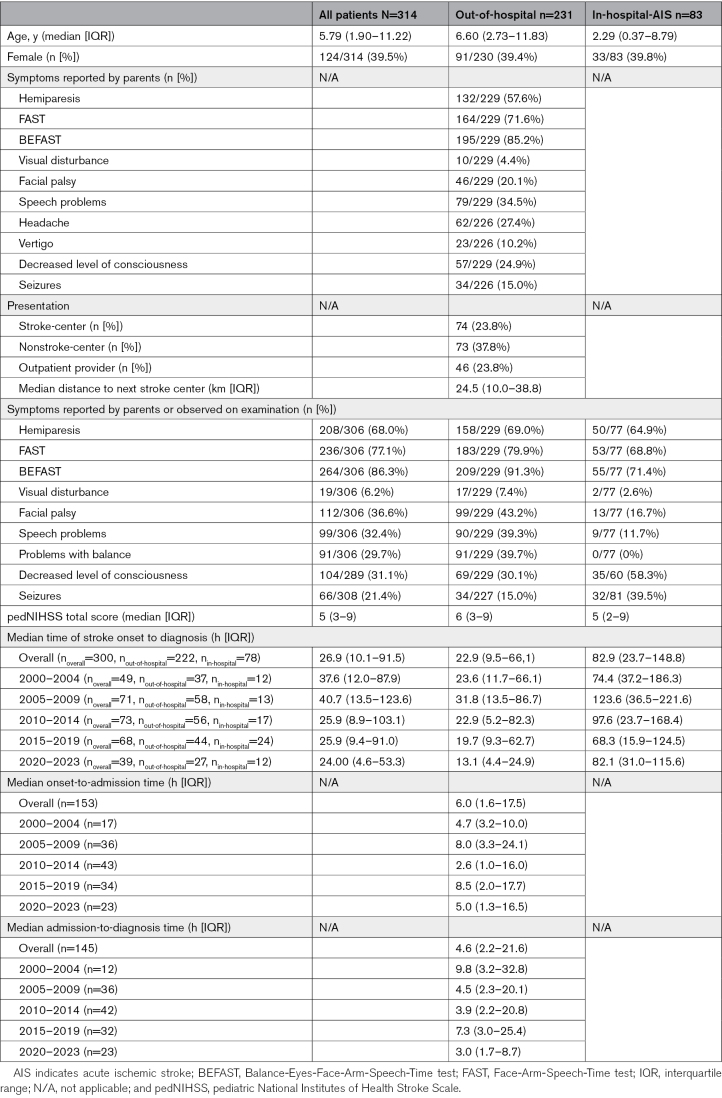
Baseline Characteristics

Children with in-hospital AIS were more likely to experience decreased level of consciousness (58.3% versus 30.1%) and epileptic seizures (39.5% versus 15.0%), whereas children with out-of-hospital AIS more frequently presented with symptoms screened by the BEFAST criteria. 91.3% of children with out-of-hospital AIS met the BEFAST criteria.

### Changes in Diagnostic Time Intervals Over the Study Period (2000–2023)

**Figure 2.**
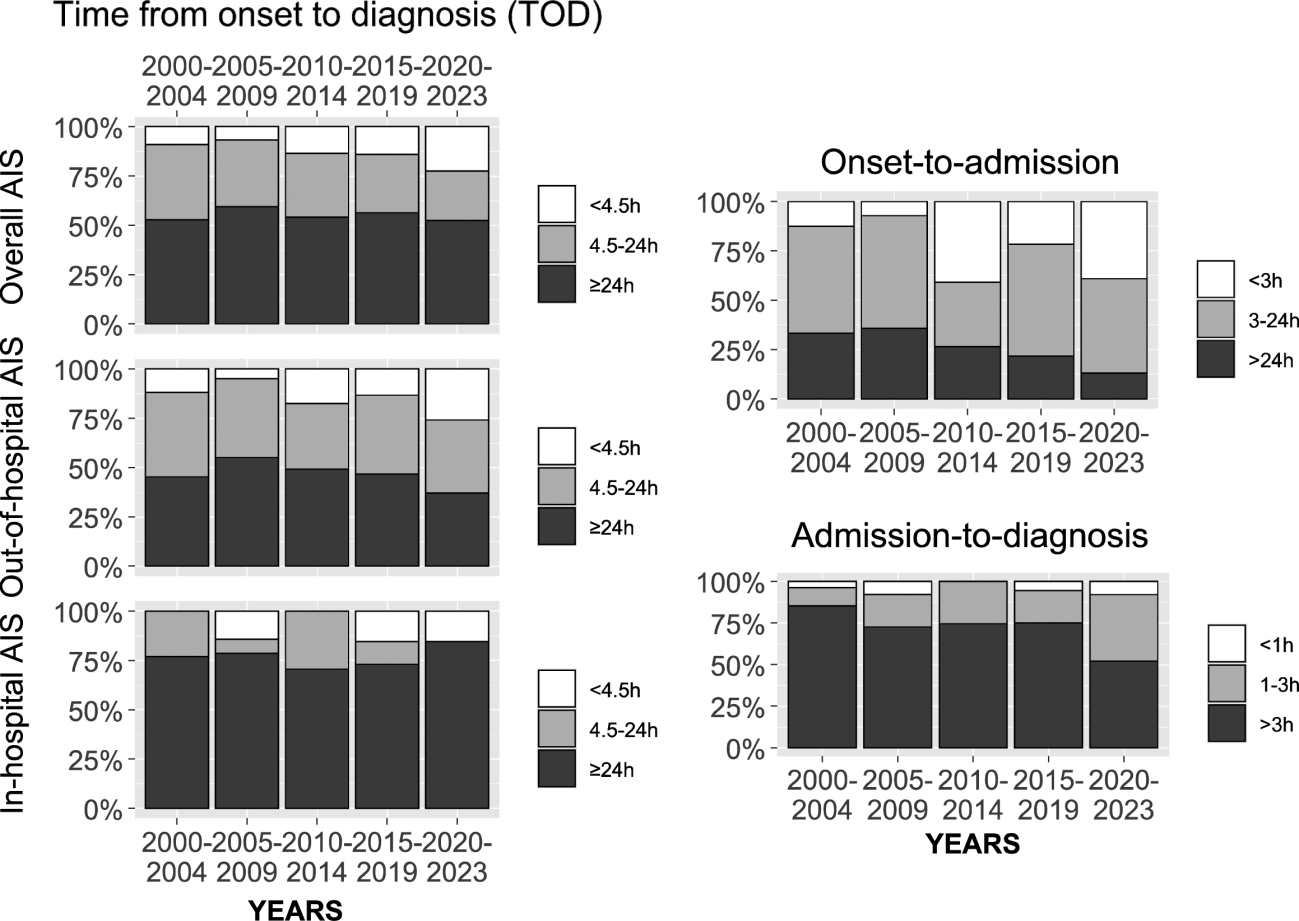
**Changes in time intervals: time from stroke onset to diagnosis (TOD), onset-to-admission, and admission-to-diagnosis time over the entire study duration.** AIS indicates acute ischemic stroke.

Changes in the distribution of categorical time intervals over the 24-year study period are illustrated in Figure 2. The median TOD in the overall study population was 26.9 hours (IQR, 10.1–91.5). It was 37.6 hours (IQR, 12.0–87.9) in the first study period (2000–2004) and 24.0 hours (IQR, 4.6–53.3) in the last study period (2020–2023). As presented in Table 2 and the Supplemental Material (Table S2), this change was not statistically significant, neither in univariate analysis (β=−0.32 [95% CI, −1.1 to 0.4]; *P*=0.41), nor when adjusting for potential confounders (β=−0.65 [95% CI, −1.43 to 0.12]; *P*=0.097). In the out-of-hospital AIS subgroup, the median TOD was 23.6 hours (IQR, 11.7–66.1) in 2000 to 2004 and 13.1 hours (IQR, 4.4–24.9) in 2020 to 2024. This change did not reach significance in the adjusted analysis (β=−0.64 [95% CI, −1.28 to 0.01]; *P*=0.053). By contrast, the adjusted analysis of in-hospital AIS cases demonstrated a statistically significant decrease in TOD (β=–4.35 [95% CI, −7.24 to −1.46]; *P*=0.004).

**Table 2.**
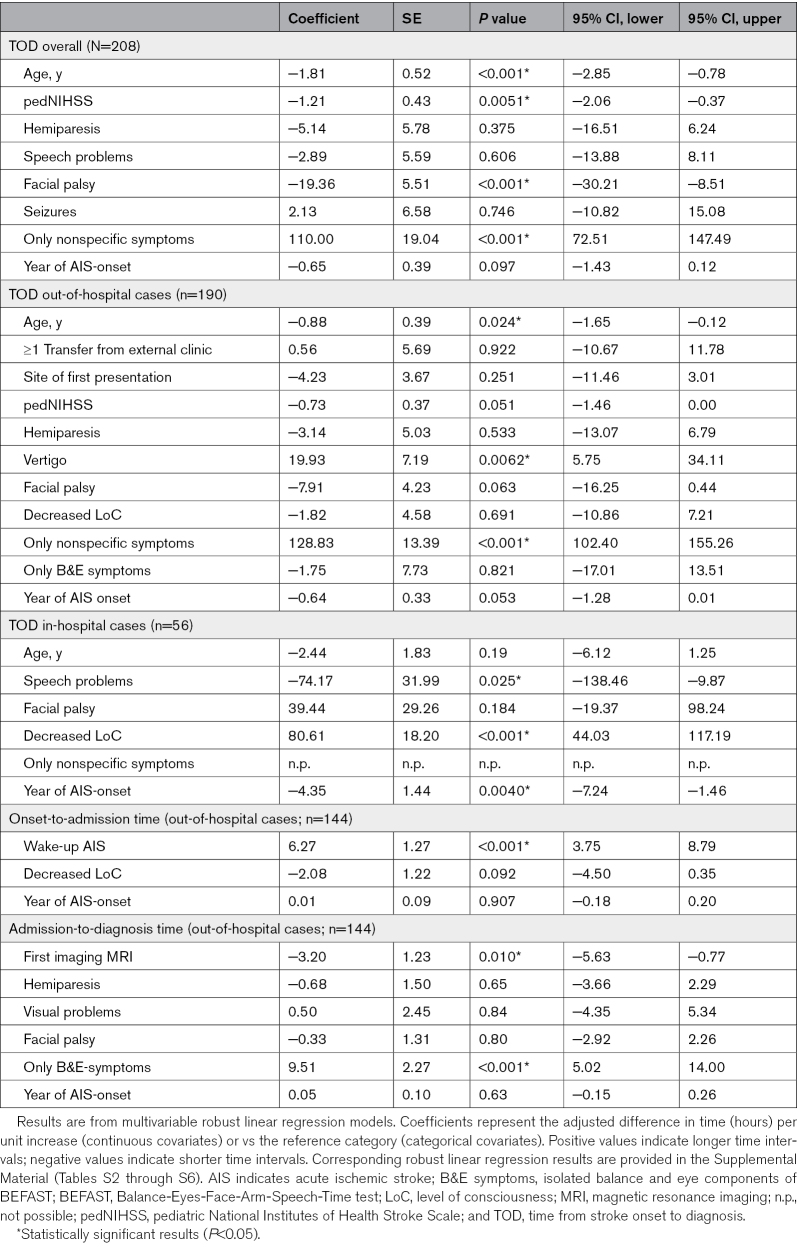
Factors Associated With Diagnostic Time Intervals (Hours) in Pediatric AIS (Multivariable Robust Linear Regression)

A post hoc exploratory analysis showed that 35 of 60 patients (58%) in the in-hospital subgroup were in a postoperative sedated state during the acute period relevant to stroke recognition. Median TOD in postoperative sedated patients (133.6 hours [IQR, 88.6–173.2]) was significantly higher (r=0.57, *P*≤0.0001) than in non-postoperative sedated cases (26.0 hours [IQR, 4.7–81.5]).

As presented in Tables 1 and 2, median onset-to-admission time fluctuated over the study periods and did not significantly decrease in multivariable models (β=0.01 [95% CI, −0.18 to 0.20]; *P*=0.91). Admission-to-diagnosis time was 9.8 hours (IQR, 3.2–32.8) in the first and 3.0 hours (IQR, 1.7–8.7) in the last study period. Again, this difference was not statistically significant after adjustment for potential confounders (β=0.05 [95% CI, −0.15 to 0.26]; *P*=0.63).

The number of diagnoses outside the thrombolysis window changed from 90.9% to 77.5% overall and from 88.1% to 74.1% in out-of-hospital cases. In the multivariable analyses, the corresponding adjusted odds ratios (overall cohort, 0.94 [95% CI, 0.88–1.00]; *P*=0.037; out-of-hospital AIS, 0.91 [95% CI, 0.84–1.00]; *P*=0.039) indicated a significant decrease in the number of diagnoses outside the thrombolysis window. In contrast, there was no significant change over time in the proportion of patients diagnosed outside the thrombectomy window across all 3 groups (see Table 3).

**Table 3.**
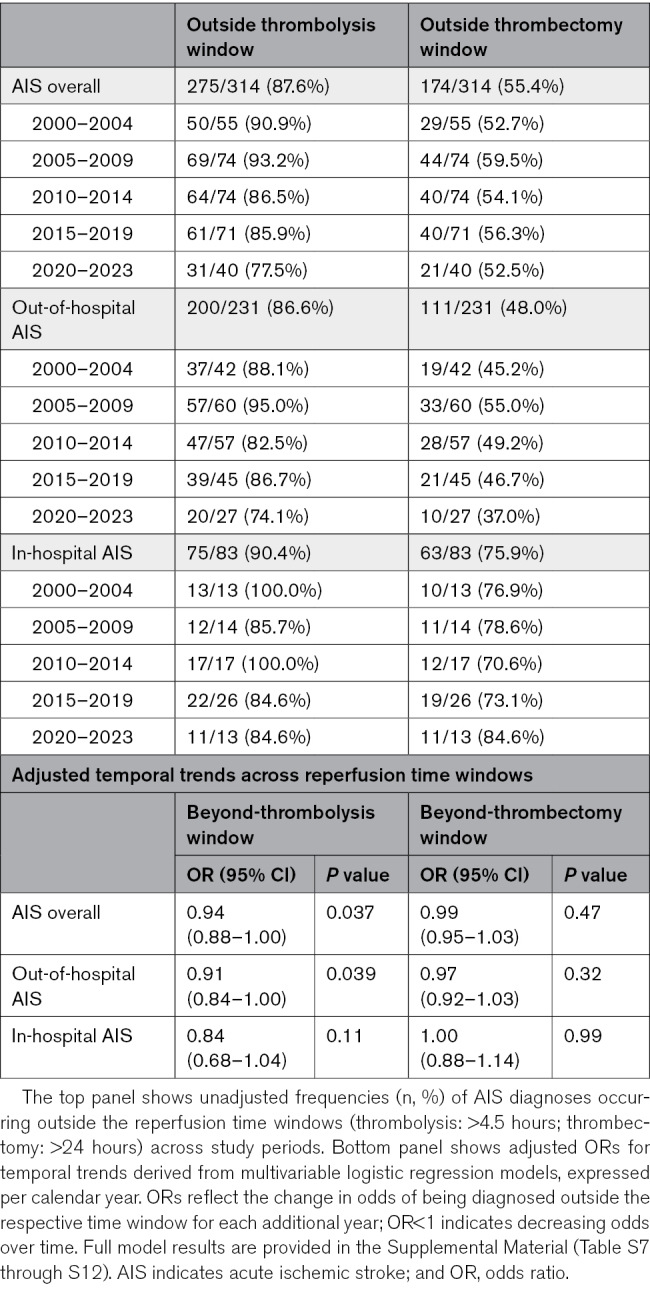
Change Over Time in the Number of Patients Diagnosed Within the Reperfusion Windows

### Factors Associated With Time Intervals

#### Patient-Related Factors

As shown in Table 2, older age was an independent predictor of shorter TOD in the overall population (β=–1.81 [95% CI, −2.85 to −0.78]; *P*<0.001) and in out-of-hospital cases (β=–0.88 [95% CI, −1.65 to −0.12]; *P*=0.024). Socioeconomic status was not associated with diagnostic delay (see Supplemental Material; Tables S2 through S6).

#### System- and Access-Related Factors

System- and access-related factors were only associated with longer diagnostic intervals in out-of-hospital AIS cases. Distance to the nearest stroke center did not affect the time intervals. However, the type of first presentation site was a determinant of diagnostic delay (see Table 4). None of the 46 children presenting first to an outpatient provider was diagnosed within the thrombolysis window. Compared with initial presentation to an outpatient provider, those presenting to a nonstroke center had significantly shorter time to diagnosis (mean difference, −155 hours, r=0.21, *P*=0.026). Presentation directly to a stroke center further reduced time (mean difference to nonstroke center presentation, −9.5 hours, r=0.19, *P*=0.044). Presentation site was also associated with longer TOD in the out-of-hospital group in univariate linear models, but didn’t remain significant after controlling for confounders (β=−4.32 [95% CI, −11.46 to 3.01]; *P*=0.25). Similarly, interhospital transfer was associated with longer TOD in univariate analysis for out-of-hospital cases, but this did not retain significance in multivariable models. Magnetic resonance imaging (MRI), as the first imaging modality, was significantly associated with shorter admission-to-diagnosis time (β=–3.20 [95% CI, −5.63 to −0.77]; *P*=0.010).

**Table 4.**
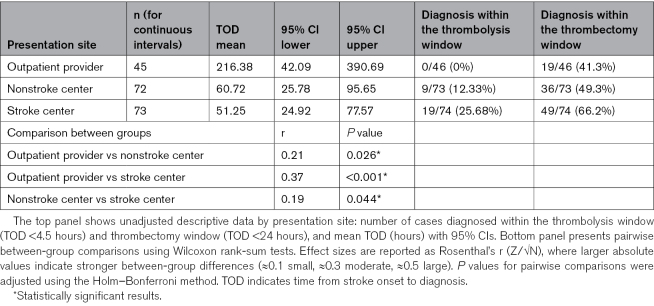
Comparison of TOD and Diagnoses Made Within Reperfusion Window Between Presentation Site for Out-of-Hospital Cases

#### Clinical Characteristics

As shown in Table 2 in the total AIS cohort higher pedNIHSS (β=−1.21 [95% CI, −2.06 to −0.37]; *P*=0.0051) and facial palsy (β=19.36 [95% CI, −30.21 to −8.51]; *P*<0.001) was independently associated with shorter TOD, whereas the presence of nonspecific symptoms (β=110.00 [95% CI, 72.51–147.49]; *P*<0.001) was linked to substantially longer TOD.

In the out-of-hospital subgroup, vertigo (β=19.93 [95% CI, 5.75–34.11]; *P*=0.0062) and the presence of nonspecific symptoms (β=128.83 [95% CI, 102.40–155.26]; *P*<0.001) were associated with longer TOD. In in-hospital patients with AIS, speech problems (β=−74.17 [95% CI, −138.46 to −9.87]; *P*=0.025) were associated with shorter TOD, whereas decreased level of consciousness (β=80.61 [95% CI, 44.03–117.19]; *P*<0.001) was linked to prolonged TOD.

Wake-up AIS (β=6.27 [95% CI, 3.75–8.79]; *P*<0.001) was associated with longer onset-to-admission time. For admission-to-diagnosis time, only the presence of the B&E symptoms from the BEFAST (β=9.51 [95% CI, 5.02–14.00]; *P*<0.001) was associated with longer delays.

### Proportion of Patients Diagnosed Within Reperfusion Windows by FAST and BEFAST Status

Among children fulfilling FAST criteria, the highest proportion received a diagnosis within the thrombolysis or thrombectomy window, followed closely by those fulfilling BEFAST criteria (see Table 5). No child presenting exclusively with B&E symptoms of the BEFAST was diagnosed within the thrombolysis window, and B&E-only–positive patients were significantly less likely to receive a diagnosis within the thrombolysis window compared with FAST-positive cases (odds ratio, 0.0; *P*=0.032).

**Table 5.**
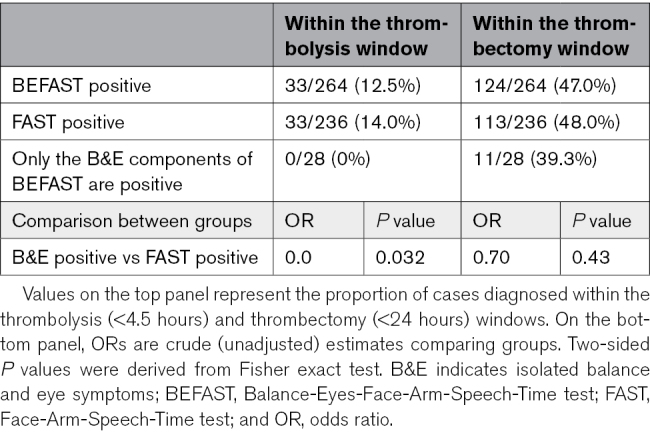
Comparison of Proportion of Diagnoses Within Reperfusion Windows Among FAST, BEFAST, and B&E-Positive Cases

No significant difference in diagnoses within the thrombectomy window was observed between B&E-only and FAST-positive patients.

## Discussion

In this nationwide Swiss pediatric stroke cohort, diagnoses within the intravenous-thrombolysis window (≤4.5 hours) increased over 24 years, largely driven by out-of-hospital AIS. Median TOD only showed a significant decrease in the in-hospital subgroup, yet this did not translate into more reperfusion-window diagnoses. In all pediatric stroke cases, 3 quarters of diagnoses were made outside the thrombolysis window, despite revascularization being one of the most effective options in eligible children.^1,2^ Single-center and regional cohorts from 4 continents reported median TODs comparable to ours for out-of-hospital AIS, particularly before 2016.^6–9,27^ Whereas prior studies reported shorter TODs for in-hospital AIS (21–23 hours), our median TOD was considerably longer (82.9 hours [IQR, 23.7–148.8]). This discrepancy may reflect methodological differences, with prior studies using symptom discovery rather than the last known well as the definition of onset in postoperative sedated patients.^6,9^ In our cohort, more than half of the in-hospital cases were postoperative and sedated, and this subgroup showed a significantly longer TOD in post hoc analysis.

Thrombolysis-window diagnoses approximated prior United Kingdom data.^7^ For the thrombectomy window, rates were also consistent with international reports.^7,8^ The previously reported high rate of 77% thrombectomy-window diagnoses in our registry for 2000 to 2006 was revised to 52.7% in the present study after re-adjudication using harmonized definitions and case-level review, thereby aligning more closely with external cohorts.^7,28^ Onset-to-admission and admission-to-diagnosis times in our study were within the wide ranges reported in previous studies.^7,9,27^ After adjustment for potential confounders, neither of those intervals showed significant improvement over time. In contrast, a Canadian cohort reported a decrease in categorical onset-to-admission time between 2009 and 2014.^20^ Differences in analytic approach and cohort composition may explain this difference. Given that our out-of-hospital TOD improvements were concentrated at ≤4.5 hours, which didn’t lead to a significant decrease in continuous TOD, shifts between categories may also not be visible when modeling onset-to-admission time continuously.

### Factors Associated With Time Intervals

Younger age was associated with longer TOD overall and in out-of-hospital AIS, likely reflecting the higher frequency of nonspecific presentations (eg, somnolence) in younger children.^29^ Socioeconomic status was not associated with timing, consistent with prior pediatric data.^20^ These findings highlight the need to raise awareness of atypical presentations in younger children among first point of contact providers. Age-tailored recognition may help reduce diagnostic delays.

System and access factors highlighted the importance of the first point of contact. Direct presentation to stroke centers minimized TOD, with longer delays at nonstroke hospitals and the longest at outpatient providers. Time from onset to reperfusion treatment, the decisive interval for reperfusion, not captured here, would likely diverge further between stroke and nonstroke centers, as hyperacute therapy is unavailable at the latter. Interhospital transfer and the distance to the next stroke center were not significantly associated with diagnostic delay. These findings mirror pediatric data from Toronto, where the site of first presentation was a dominant system and access factor.^9^ In contrast, adult stroke has higher awareness and direct pathways to stroke centers, making distance and transfer key factors for delay.^30^ Our findings support caregiver and first point of contact provider awareness campaigns and emphasize prehospital triage of suspected pediatric stroke to specialized centers. The latter was recently shown to reduce time from stroke onset to reperfusion treatment in a French study.^2^

Cerebral MRI as an initial imaging modality was associated with shorter admission-to-diagnosis time, consistent with MRI’s superiority in pediatric stroke diagnostics compared with computed tomography.^31^ Yet this advantage did not materially reduce TOD across presentation groups, underscoring that upstream processes dominate overall delay. Although we could not directly assess the effectiveness of structured in- and prehospital stroke protocols, which are known to reduce admission-to-diagnosis time, our findings indirectly highlight their importance: the association between MRI-first and shorter admission-to-diagnosis time illustrates that key components of these protocols, such as prehospital triage to MRI-capable centers and rapid in-hospital MRI access, are crucial for timely diagnosis.^19^

Clinical factors were the key determinant of diagnostic delay in the in-hospital group. Consistent with adult data, the strongest association with delayed diagnosis was observed in cases with decreased level of consciousness.^32^ This likely reflects competing critical-care priorities and the challenge of recognizing superimposed focal deficits. Speech disturbance correlated with shorter TOD, probably reflecting ascertainment bias (only evaluable in awake, nonintubated children) rather than a direct effect. These findings underscore the need for systematic surveillance of high-risk patients for stroke, many of whom are children following cardiac interventions. The recently proposed postprocedural stroke screening tool may support this effort.^33^ In the overall cohort, higher pedNIHSS was associated with shorter TOD, consistent with previous reports, reflecting earlier recognition of overt symptoms.^9^ As already shown in adult data, facial palsy remained independently associated with shorter TOD in our cohort.^34^ This may relate to its association with anterior-circulation strokes reported in pediatric studies and with large-vessel occlusion described in adults, both potentially linked to shorter diagnostic delays.^35–37^

As already shown, posterior circulation symptoms were consistently associated with diagnostic delay.^9,38^ BEFAST positivity was high in out-of-hospital AIS cases (91%), but markedly lower in in-hospital AIS. Within BEFAST, cases presenting with isolated posterior stroke components (B&E symptoms) were rarely diagnosed within 24 hours, never within 4.5 hours, and were strongly associated with prolonged admission-to-diagnosis time. Vertigo, as a posterior stroke sign, is also related to longer TOD in out-of-hospital cases. Consistent with prior work, our data suggest that recognition difficulty in emergency settings, rather than caregiver delay, is the main barrier for posterior strokes.^9^ These findings support BEFAST as a reliable tool for pediatric stroke recognition, although its awareness may be limited among first point of contact providers.^39^ They further highlight the need to incorporate posterior stroke signs, emphasizing the B&E part of the BEFAST into training for first point of contact providers.

Wake-up AIS was the only independent factor associated with longer onset-to-admission time, likely reflecting the use of the last known well as the stroke onset anchor when symptoms were first recognized on awakening. No further system-related drivers of caregiver delay were identified, suggesting that efforts to raise caregiver awareness should remain broad. Yet a 6.0-hour median onset-to-admission time limits intravenous thrombolysis opportunities, highlighting the need to accelerate prehospital care. However, increasing awareness and activation of pre- and intrahospital stroke pathways may also raise the proportion of stroke mimics undergoing MRI, which in children often requires sedation and therefore carries additional risks for those ultimately not diagnosed with stroke. This underscores the need to identify reliable biomarkers to improve triage accuracy in suspected pediatric stroke.

### Strengths and Limitations of This Study

Strengths include a nationwide cohort spanning tertiary and smaller pediatric hospitals, harmonized definitions with case-level re-adjudication, and the distinction between parent-reported and clinician-observed symptoms. Limitations include the retrospective design with missing time points (up to 37% for onset-to-admission time) due to incomplete documentation of key datapoints, which is common in long-running clinical registries^40^ and increases the risk for a potential selection bias. Future registry improvements should therefore prioritize standardized, required time fields and streamlined electronic data entry to reduce missingness. Subgroup analyses were limited by a relatively small sample, which may have reduced statistical power to detect temporal trends or associations. Finally, we used TOD as a proxy for timely diagnosis within reperfusion windows. However, for initial presentations to non-reperfusion-capable hospitals, potential transfer-related delays were not captured, potentially underestimating the clinically relevant time interval in these patients.

### Conclusions

The proportion of diagnoses within ≤4.5 hours increased over 2 decades in Swiss pediatric AIS, largely driven by out-of-hospital cases. After adjustment, in-hospital cases showed a significant decrease in continuous TOD, which did not translate into more reperfusion-window diagnoses. Because posterior stroke symptoms and initial contact outside stroke centers remain the dominant barriers, priority actions are training first point of contact providers to recognize BEFAST B&E signs, direct-to-center pathways, and standardized pediatric AIS protocols (MRI first), as well as caregiver awareness campaigns.

## ARTICLE INFORMATION

### Acknowledgments

The authors thank Maria Regenyi for her support in data retrieval from the Swiss Neuropediatric Stroke Registry. Finally, the authors acknowledge the use of ChatGPT for language polishing of this work.

### Disclosures

None.

### Supplemental Material

Tables S1–S12

Figures S1–S5

STROBE Statement

## Supplementary Material


