# Supplementary material for: Diagnostic Delays in Pediatric Acute Ischemic Stroke: 24-Year Trends and Contributing Factors in Switzerland
Source: Stroke. 2026 May 1;57(7):2001–12. doi: 10.1161/STROKEAHA.125.054402 (PMC13281979; doi:10.1161/STROKEAHA.125.054402)
Supplement: Supplementary file 1 [file str-57-2001-s001.pdf]

Supplementary Material

Table S1: Assessment of normality

	Skewness	Sharpiro-Wilk-Test p-Value
Time from onset to diagnosis (TOD)	6.6	**<0.0001**
TOD in-hospital	5.7	**<0.0001**
TOD out-of-hospital	6.9	**<0.0001**
Onset-to-admission time	7.6	**<0.0001**
Admission-to-diagnosis time	4.5	**<0.0001**

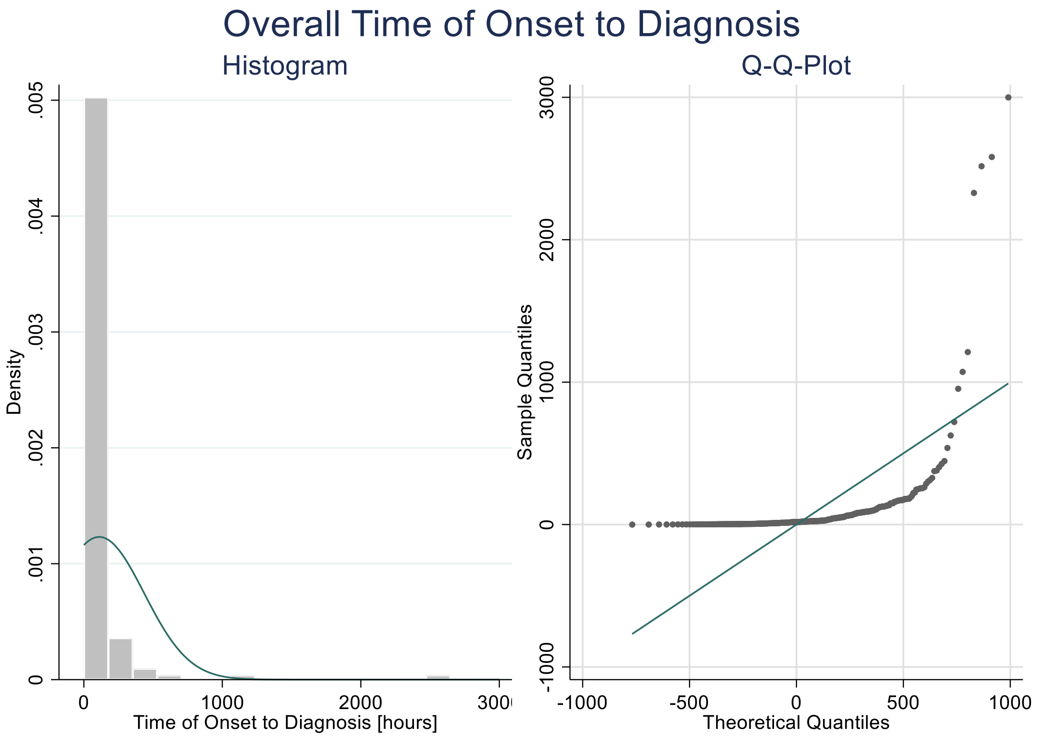

Figure S1: Assessment of normality for overall continuous Time of Onset to diagnosis. Histograms with overlaid normal distribution curves are shown on the left; corresponding quantile–quantile (Q–Q) plots are shown on the right.

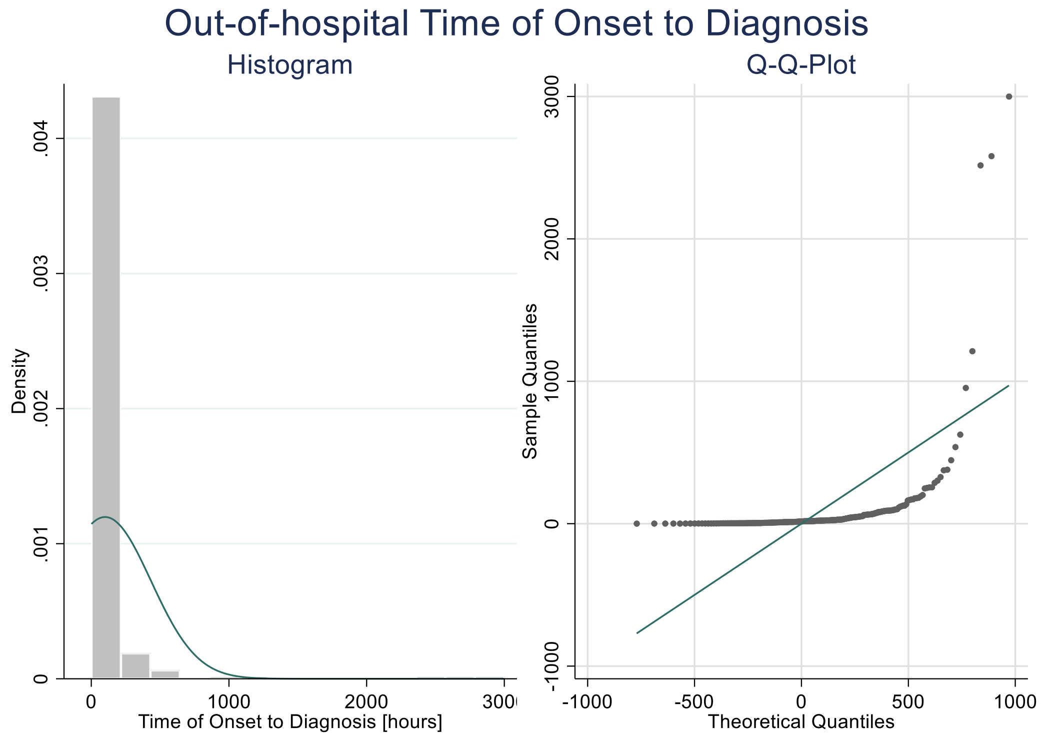

Figure S2: Assessment of normality for continuous Time of Onset to diagnosis in the out-of-hospital subgroup. Histograms with overlaid normal distribution curves are shown on the left; corresponding quantile–quantile (Q–Q) plots are shown on the right.

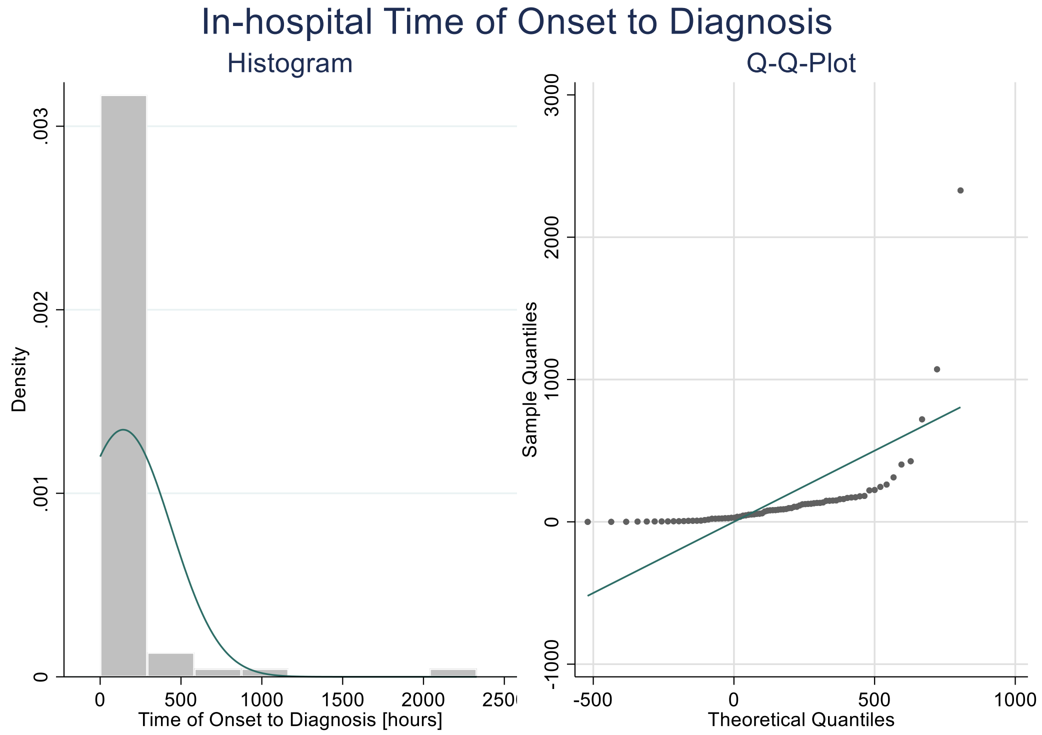

Figure S3: Assessment of normality for continuous Time of Onset to diagnosis in the in-hospital subgroup. Histograms with overlaid normal distribution curves are shown on the left; corresponding quantile–quantile (Q–Q) plots are shown on the right.

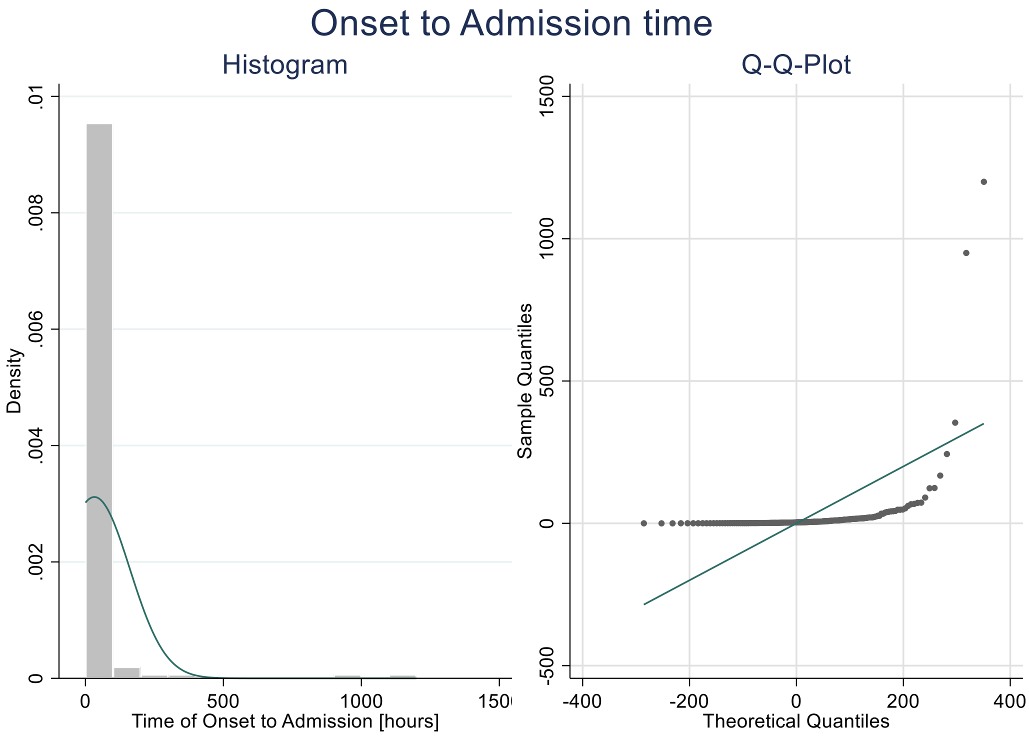

Figure S4: Assessment of normality for continuous Time of Onset to Admission. Histograms with overlaid normal distribution curves are shown on the left; corresponding quantile–quantile (Q–Q) plots are shown on the right.

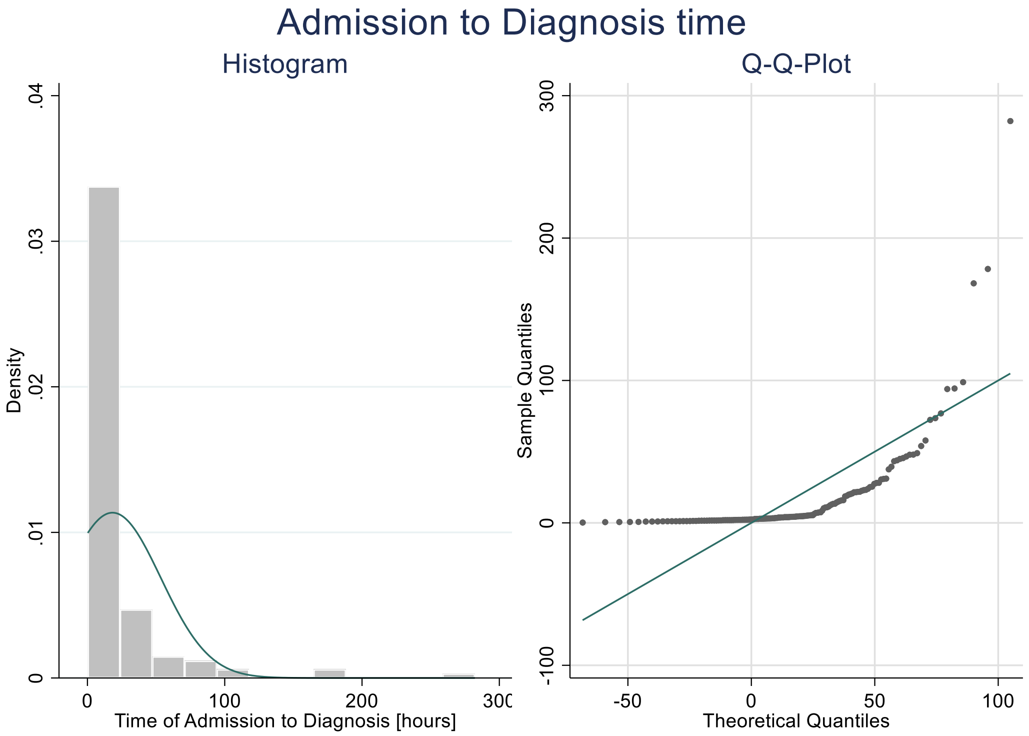

Figure S5: Assessment of normality for continuous Time of Admission to Diagnosis. Histograms with overlaid normal distribution curves are shown on the left; corresponding quantile–quantile (Q–Q) plots are shown on the right.

Table S2: Time from onset to diagnosis (TOD) – Overall AIS

	Univariate linear model				
	**Coef.**	**SE**	**95% CI-Lower**	**95% CI-Upper**	**p**
**Age in years**	-1.93	0.50	-2.91	-0.96	**0.00011**
**Manifest. on weekend**	-5.59	5.57	-16.51	5.32	0.32
**Swiss-SEP**	0.18	0.23	-0.26	0.63	0.41
**First image MRI**	-3.09	4.81	-12.52	6.35	0.52
**pedNIHSS**	-1.55	0.42	-2.36	-0.73	**0.00020**
**Hemiparesis**	-10.26	4.83	-19.74	-0.79	**0.034**
**Headache**	-10.55	6.27	-22.84	1.74	0.092
**Vertigo**	10.21	8.73	-6.91	27.32	0.24
**Visual problems**	-5.57	9.99	-25.16	14.01	0.58
**Speech problems**	-14.77	5.54	-25.62	-3.92	**0.0076**
**Facial palsy**	-22.23	5.25	-32.52	-11.95	**0.000023**
**Balance problems**	-5.24	5.25	-15.53	5.05	0.32
**Decreased LoC**	-6.14	4.89	-15.72	3.45	0.21
**Seizures**	12.29	5.98	0.56	24.02	**0.040**
**Only non-specific symptoms**	135.33	18.20	99.66	171.01	**<0.000001**
**Only B&E-symptoms**	7.42	8.46	-9.16	23.99	0.38
**Year of stroke**	-0.32	0.38	-1.06	0.43	0.41

Univariate robust linear regression analyses for time from stroke onset to diagnosis in the whole cohort. “n.p.” indicates that regression was not possible due to insufficient case numbers. LoC = Level of Consciousness; Swiss-SEP = Swiss Socioeconomic Position Index; PedNIHSS = Pediatric National Institutes of Health Stroke Scale. Statistically significant results (p < 0.05) are shown in bold.

Table S3: Time from onset to diagnosis (TOD) - Out-of-hospital AIS

	Univariate linear model				
	**Coef.**	**SE**	**95% CI-Lower**	**95% CI-Upper**	**p**
**Age in years**	-0.77	0.39	-1.53	-0.01	**0.046**
**Wakeup-AIS**	5.64	3.74	-1.68	12.97	0.13
**Manifest. on weekend**	0.59	4.28	-7.80	8.97	0.89
**≥1 transfer**	7.18	3.46	0.40	13.96	**0.038**
**Presentation**	-6.47	2.09	-10.57	-2.38	**0.0020**
**Distance to next stroke center**	-0.08	0.11	-0.30	0.13	0.44
**Swiss-SEP**	0.01	0.20	-0.38	0.39	0.97
**First image MRI**	-3.76	3.79	-11.19	3.66	0.32
**pedNIHSS**	-0.80	0.32	-1.43	-0.17	**0.013**
**Hemiparesis**	-8.60	4.05	-16.53	-0.67	**0.034**
**Headache**	-0.66	4.42	-9.31	8.00	0.88
**Vertigo**	16.69	6.43	4.09	29.28	**0.0094**
**Visual problems**	3.81	7.65	-11.18	18.81	0.62
**Speech problems**	-2.09	3.98	-9.90	5.71	0.60
**Facial palsy**	-8.91	3.82	-16.40	-1.43	**0.020**
**Balance problems**	3.57	3.85	-3.97	11.11	0.35
**Decreased LoC**	-8.89	3.61	-15.97	-1.80	**0.014**
**Seizures**	-2.58	5.40	-13.17	8.01	0.63
**Only non-specific symptoms**	142.41	14.36	114.28	170.55	**<0.000001**
**Only B&E-symptoms**	13.11	6.35	0.66	25.56	**0.039**
**Year of stroke**	-0.73	0.31	-1.33	-0.12	**0.018**

Univariate robust linear regression analyses for time from stroke onset to diagnosis in out-of-hospital AIS cases. “n.p.” indicates that regression was not possible due to insufficient case numbers. LoC = Level of Consciousness; Swiss-SEP = Swiss Socioeconomic Position Index; PedNIHSS = Pediatric National Institutes of Health Stroke Scale. Statistically significant results (p < 0.05) are shown in bold.

Table S4: Time from onset to diagnosis (TOD) - In-hospital AIS

	Univariate linear model				
	Coef.	SE	95% CI-Lower	95% CI-Upper	p
**Age in years**	-3.97	1.62	-7.14	-0.80	**0.014**
**Manifest. on weekend**	-34.38	24.70	-82.79	14.03	0.16
**Swiss-SEP**	-1.22	0.75	-2.69	0.24	0.10
**First image MRI**	0.46	18.01	-34.85	35.77	0.98
**pedNIHSS**	-0.66	1.62	-3.83	2.51	0.68
**Hemiparesis**	-8.73	20.48	-48.87	31.40	0.67
**Headache**	-75.44	46.21	-166.01	15.13	0.10
**Vertigo**	n.p.	n.p.	n.p.	n.p.	n.p.
**Visual problems**	-80.05	58.50	-194.71	34.61	0.17
**Speech problems**	-75.56	29.06	-132.51	-18.60	**0.0093**
**Facial palsy**	-61.93	23.71	-108.40	-15.46	**0.0090**
**Decreased LoC**	83.48	16.86	50.42	116.53	**0.0000007**
**Seizures**	29.36	18.15	-6.22	64.94	0.11
**Only non-specific Symptoms**	638.78	79.50	482.97	794.59	**<0.000001**
**Only B&E-symptoms**	-59.02	83.49	-222.67	104.63	0.48
**Year of stroke**	-1.60	1.39	-4.32	1.11	0.25

Univariate robust linear regression analyses for time from stroke onset to diagnosis in in-hospital AIS cases. "n.p." indicates that regression was not possible due to insufficient case numbers. Swiss-SEP = Swiss Socioeconomic Position Index; PedNIHSS = Pediatric National Institutes of Health Stroke Scale, . LoC = Level of Consciousness. Statistically significant results (p < 0.05) are shown in bold.

Table S5: Onset-to admission time

	Univariate linear model				
	Coef.	SE	95% CI-Lower	95% CI-Upper	p
**Age in years**	-0.06	0.12	-0.30	0.18	0.62
**Manifest. on weekend**	1.00	1.32	-1.60	3.59	0.45
**Wake-up stroke**	7.12	1.15	4.86	9.38	**<0.000001**
**Swiss-SEP**	0.00	0.06	-0.12	0.12	0.98
**pedNIHSS**	-0.15	0.10	-0.34	0.05	0.15
**Hemiparesis**	-1.32	1.18	-3.65	1.00	0.26
**Headache**	1.01	1.32	-1.58	3.60	0.45
**Vertigo**	1.55	1.91	-2.19	5.28	0.42
**Visual problems**	-2.27	3.11	-8.35	3.82	0.47
**Speech problems**	-0.44	1.27	-2.93	2.05	0.73
**Facial palsy**	-0.51	1.56	-3.56	2.54	0.74
**Balance problems**	1.04	1.28	-1.46	3.55	0.41
**Decreased LoC**	-3.54	1.42	-6.33	-0.75	**0.013**
**Seizures**	-1.62	1.70	-4.96	1.72	0.34
**Only B&E-symptoms**	1.60	1.88	-2.09	5.28	0.40
**Year of stroke**	0.05	0.10	-0.14	0.25	0.60

Univariate robust linear regression analyses for time of stroke onset to admission for out-of-hospital AIS. "n.p." indicates that regression was not possible due to insufficient case numbers. Swiss-SEP = Swiss Socioeconomic Position Index; PedNIHSS = Pediatric National Institutes of Health Stroke Scale, . LoC = Level of Consciousness. Bold values indicate statistically significant results (p < 0.05)

Table S6: Admission-to-diagnosis time

	Univariate linear model				
	**Coef.**	**SE**	**95% CI-Lower**	**95% CI-Upper**	**p**
**Age in years**	-0.04	0.08	-0.19	0.11	0.64
**Manifestation on weekend**	-1.31	0.76	-2.80	0.18	0.086
**≥1 transfer**	0.48	0.85	-1.20	2.15	0.58
**Presentation**	-0.58	0.55	-1.66	0.49	0.29
**1st image MRI**	-4.04	1.37	-6.72	-1.36	0.0032
**Swiss-SEP**	0.03	0.04	-0.06	0.11	0.53
**pedNIHSS**	0.00	0.07	-0.14	0.14	0.97
**Hemiparesis**	-3.67	0.95	-5.54	-1.80	**0.00012**
**Headache**	2.04	1.12	-0.15	4.24	0.068
**Vertigo**	2.03	1.35	-0.62	4.68	0.13
**Visual problems**	7.32	1.77	3.84	10.79	**0.000037**
**Speech problems**	-0.36	0.83	-2.00	1.27	0.66
**Facial palsy**	-1.93	0.75	-3.40	-0.47	**0.0098**
**Balance problems**	0.19	0.84	-1.46	1.84	0.82
**Decreased LoC**	-0.13	0.92	-1.94	1.68	0.89
**Seizures**	0.78	1.19	-1.56	3.11	0.52
**Only B&E-symptoms**	12.00	1.17	9.72	14.29	**<0.000001**
**Only symptoms before presentation**	-1.35	1.86	-4.99	2.29	0.47
**Year of stroke**	-0.08	0.07	-0.22	0.06	0.27

Univariate regression analyses for Time of admission to diagnosis for out-of-hospital AIS
Shown are the results of univariate logistic and linear regression models assessing associations between clinical covariates and time to diagnosis (categorical and continuous). "n.p." indicates that regression was not possible due to insufficient case numbers. Swiss-SEP = Swiss Socioeconomic Position Index; PedNIHSS = Pediatric National Institutes of Health Stroke Scale, . LoC = Level of Consciousness. Bold values indicate statistically significant results (p < 0.05).

Table S7: Time from onset to Diagnosis (TOD) - overall AIS thrombolysis window

	Descriptive		Univariate logistic model				
	<4.5h Cont.: mean [min;max] Categ.: ratios(%)	>4.5h Cont.: mean [min;max] Categ.: ratios(%)	OR	SE	95% CI-Lower	95% CI-Upper	p
**Age in years**	9.76 [0.46;15.91]	6.15 [.11;15.99]	0.87	0.03	0.81	0.93	**0.000072**
**Manifest. on weekend**	12/39 (31%)	66/275 (24%)	0.71	0.27	0.34	1.48	0.36
**Swiss-SEP**	60.78 [31.82;77.45]	61.86 [28.49;88.62]	1.01	0.02	0.98	1.04	0.60
**First image MRI**	22/39 (56%)	125/275 (45%)	0.61	0.22	0.31	1.22	0.16
**pedNIHSS**	9.54 [0;30]	6.18 [0;40]	0.92	0.02	0.88	0.97	**0.0020**
**Hemiparesis**	31/39 (79%)	177/275 (64%)	0.51	0.21	0.22	1.15	0.10
**Headache**	7/39 (18%)	59/275 (21%)	1.28	0.57	0.54	3.06	0.57
**Vertigo**	1/39 (3%)	24/275 (9%)	3.72	3.85	0.49	28.32	0.20
**Visual problems**	2/39 (5%)	17/275 (6%)	1.26	0.97	0.28	5.67	0.77
**Speech problems**	19/39 (49%)	80/275 (29%)	0.45	0.16	0.23	0.89	**0.022**
**Facial palsy**	22/39 (56%)	90/275 (33%)	0.39	0.14	0.20	0.78	**0.0073**
**Balance problems**	11/39 (28%)	80/275 (29%)	1.09	0.41	0.52	2.29	0.82
**Decreased LoC**	21/39 (54%)	74/275 (27%)	0.33	0.11	0.17	0.65	**0.0014**
**Seizures**	7/39 (18%)	59/275 (21%)	1.28	0.57	0.54	3.06	0.57
**Only non-specific Symptoms**	0/39 (0%)	5/275 (2%)	n.p.	n.p.	n.p.	n.p.	n.p.
**Only B&E-symptoms**	0/39 (0%)	28/275 (10%)	n.p.	n.p.	n.p.	n.p.	n.p.
**Year of stroke**	2013.36 [2001;2023]	2011.05 [2000;2023]	0.94	0.03	0.90	1.00	**0.038**

Multivariate model	OR	SE	p	95% CI Lower	95% CI Upper
**Age in years**	0.87	0.04	**0.0011**	0.80	0.95
**pedNIHSS**	0.95	0.03	0.07	0.89	1.00
**Speech Problems**	0.63	0.26	0.264	0.28	1.41
**Facial palsy**	0.35	0.15	**0.011**	0.16	0.79
**Decreased LoC**	0.43	0.18	**0.045**	0.19	0.98
**Year of stroke**	0.94	0.03	**0.037**	0.88	1.00

Above: univariate, below: multivariate logistic regression analyses for the whole cohort and the thrombolysis window. "n.p." indicates that regression was not possible due to insufficient case numbers. Swiss-SEP = Swiss Socioeconomic Position Index; PedNIHSS = Pediatric National Institutes of Health Stroke Scale; LoC = Level of Consciousness. Statistically significant results (p < 0.05) are shown in bold.

Table S8: Time from onset to diagnosis (TOD) - Out-of-hospital AIS thrombolysis window

	Descriptive		Univariate logistic model				
	<4.5h Cont.: mean [min;max] Categ.: ratios(%)	>4.5h Cont.: mean [min;max] Categ.: ratios(%)	OR	SE	95% CI-Lower	95% CI-Upper	p
**Age in years**	9.84 [0.46;15.58]	6.87 [0.18;15.99]	0.88	0.04	0.81	0.96	**0.0023**
**Wakeup-AIS**	0/31 (0%)	42/200 (21%)	n.p.	n.p.	n.p.	n.p.	n.p.
**Manifest. on weekend**	11/31 (35%)	55/200 (28%)	0.69	0.28	0.31	1.53	0.36
**≥1 transfer**	7/31 (23%)	93/200 (47%)	3.72	1.72	1.50	9.22	**0.0046**
**Presentation**	9/31 (29%)	64/200 (32%)	0.27	0.10	0.13	0.54	**0.00029**
**Distance to next stroke center**	24.63 [0.52;63.01]	27.02 [0.49;85.10]	1.01	0.01	0.98	1.03	0.54
**Swiss-SEP**	59.74 [39.37;77.45]	61.65 [35.13;88.62]	1.02	0.02	0.98	1.06	0.38
**First image MRI**	17/31 (55%)	90/200 (45%)	0.64	0.25	0.29	1.38	0.25
**pedNIHSS**	9.87 [0;30]	6.23 [0;40]	0.92	0.03	0.87	0.97	**0.0027**
**Hemiparesis**	25/31 (81%)	133/200 (67%)	0.49	0.24	0.19	1.26	0.14
**Headache**	5/31 (16%)	57/200 (29%)	2.13	1.09	0.78	5.83	0.14
**Vertigo**	1/31 (3%)	22/200 (11%)	3.79	3.95	0.49	29.20	0.20
**Visual problems**	1/31 (3%)	16/200 (8%)	2.64	2.77	0.34	20.63	0.36
**Speech problems**	14/31 (45%)	76/200 (38%)	0.76	0.29	0.35	1.62	0.47
**Facial palsy**	17/31 (55%)	82/200 (41%)	0.58	0.23	0.27	1.25	0.16
**Balance problems**	11/31 (35%)	80/200 (40%)	1.23	0.50	0.56	2.71	0.60
**Decreased LoC**	19/31 (61%)	50/200 (25%)	0.21	0.09	0.10	0.47	**0.00013**
**Seizures**	5/31 (16%)	29/200 (15%)	0.90	0.48	0.32	2.54	0.85
**Only non-specific symptoms**	0/31 (0%)	4/200 (2%)	n.p.	n.p.	n.p.	n.p.	n.p.
**Only B&E-symptoms**	0/31 (0%)	26/200 (13%)	n.p.	n.p.	n.p.	n.p.	n.p.
**Year of Stroke**	2012.84 [2001;2023]	2010.51 [2000;2023]	0.94	0.03	0.89	1.00	0.059

Multivariate model

	OR	SE	p	95% CI Lower	95% CI Upper
**Age in years**	0.85	0.04	**0.0023**	0.77	0.94
**≥1 Transfer from external clinic**	0.67	0.51	0.602	0.15	2.96
**Site of 1st presentation**	0.17	0.11	**0.0057**	0.05	0.60
**pedNIHSS**	0.93	0.04	0.069	0.86	1.01
**Decreased LoC**	0.40	0.22	0.089	0.14	1.15
**Year of Stroke**	0.91	0.04	**0.039**	0.84	1.00

Above: univariate, below: multivariate logistic regression analyses for out-of-hospital AIS and the thrombolysis window. "n.p." indicates that regression was not possible due to insufficient case numbers. Swiss-SEP = Swiss Socioeconomic Position Index; PedNIHSS = Pediatric National Institutes of Health Stroke Scale; LoC = Level of Consciousness. Statistically significant results (p < 0.05) are shown in bold.

Table S9: Time from onset to diagnosis (TOD) – In-hospital AIS thrombolysis window

	Descriptive		Univariate logistic model				
	**<4.5h Cont.: mean [min;max] Categ.: ratios(%)**	**>4.5h Cont.: mean [min;max] Categ.: ratios(%)**	**OR**	**SE**	**95% CI-Lower**	**95% CI-Upper**	**p**
**Age in years**	9.48 [2.13;15.91]	4.24 [0.11;15.9]	0.84	0.06	0.73	0.96	**0.013**
**Manifest. on weekend**	1/8 (13%)	11/75 (15%)	1.20	1.34	0.13	10.76	0.87
**Swiss-SEP**	64.17 [31.82;77.27]	62.42 [28.49;87.52]	0.99	0.03	0.93	1.05	0.72
**First image MRI**	5/8 (63%)	35/75 (47%)	0.53	0.40	0.12	2.36	0.40
**pedNIHSS**	7.83 [2;19]	6.05 [0;25]	0.96	0.06	0.84	1.08	0.49
**Hemiparesis**	6/8 (75%)	44/75 (59%)	0.59	0.50	0.11	3.13	0.53
**Headache**	2/8 (25%)	2/75 (3%)	0.08	0.09	0.01	0.71	**0.023**
**Vertigo**	0/8 (0%)	2/75 (3%)	n.p.	n.p.	n.p.	n.p.	n.p.
**Visual problems**	1/8 (13%)	1/75 (1%)	0.10	0.15	0.01	1.83	0.12
**Speech problems**	5/8 (63%)	4/75 (5%)	0.04	0.03	0.01	0.21	**0.00022**
**Facial palsy**	5/8 (63%)	8/75 (11%)	0.08	0.06	0.02	0.39	**0.0020**
**Decreased LoC**	0/8 (0%)	35/75 (47%)	1.00				
**Seizures**	2/8 (25%)	30/75 (40%)	2.09	1.78	0.40	11.08	0.39
**Only non-specific symptoms**	0/8 (0%)	1/75 (1%)	n.p.	n.p.	n.p.	n.p.	n.p.
**Only B&E-symptoms**	0/8 (0%)	2/75 (3%)	n.p.	n.p.	n.p.	n.p.	n.p.
**Year of Stroke**	2015.38 [2006;2022]	2012.48 [2000;2023]	0.93	0.06	0.82	1.05	0.25

Multivariate model

	**OR**	**SE**	**p**	**95% CI Lower**	**95% CI Upper**
**Age in years**	1.00	0.13	0.999	0.77	1.30
**Headache**	0.05	0.11	0.159	0.00	3.20
**Speech problems**	0.01	0.02	**0.012**	0.00	0.40
**Facial palsy**	0.33	0.40	0.354	0.03	3.41
**Year of Stroke**	0.84	0.09	0.108	0.68	1.04

Above: univariate, below: multivariate logistic regression analyses for in-hospital AIS and the thrombolysis window. "n.p." indicates that regression was not possible due to insufficient case numbers. Swiss-SEP = Swiss Socioeconomic Position Index; PedNIHSS = Pediatric National Institutes of Health Stroke Scale; LoC = Level of Consciousness. Statistically significant results (p < 0.05) are shown in bold.

Table S10: Time Onset to Diagnosis (TOD) - OVERALL AIS Thrombectomy-Window

	Descriptive		Univariate logistic model				
	<24h mean [min;max] Categ.: ratios(%)	>24h Cont.: mean [min;max] Categ.: ratios(%)	OR	SE	95% CI-Lower	95% CI-Upper	p
**Age in years**	7.71 [0.22;15.95]	5.71 [0.11;15.99]	0.92	0.02	0.88	0.97	**0.00059**
**Manifest. on weekend**	39/140 (28%)	39/174 (22%)	0.75	0.20	0.45	1.25	0.27
**Swiss-SEP**	62.15 [31.82;87.52]	61.39 [28.49;88.62]	0.99	0.01	0.97	1.01	0.56
**First image MRI**	66/140 (47%)	81/174 (47%)	0.96	0.22	0.61	1.50	0.86
**pedNIHSS**	7.99 [0;40]	5.45 [0;27]	0.92	0.02	0.88	0.96	**0.00029**
**Hemiparesis**	103/140 (74%)	105/174 (60%)	0.57	0.14	0.35	0.93	**0.024**
**Headache**	37/140 (26%)	29/174 (17%)	0.55	0.15	0.32	0.96	**0.034**
**Vertigo**	7/140 (5%)	18/174 (10%)	2.18	1.01	0.88	5.39	0.090
**Visual problems**	10/140 (7%)	9/174 (5%)	0.72	0.34	0.29	1.84	0.50
**Speech problems**	55/140 (39%)	44/174 (25%)	0.54	0.13	0.33	0.87	**0.011**
**Facial palsy**	70/140 (50%)	42/174 (24%)	0.32	0.08	0.20	0.52	**0.0000048**
**Balance problems**	48/140 (34%)	43/174 (25%)	0.65	0.16	0.39	1.06	0.081
**Decreased LoC**	48/140 (34%)	47/174 (27%)	0.73	0.18	0.45	1.18	0.20
**Seizures**	23/140 (16%)	43/174 (25%)	1.67	0.48	0.95	2.93	0.077
**Only non-specific symptoms**	1/140 (1%)	4/174 (2%)	3.31	3.72	0.37	29.92	0.29
**Only B&E-symptoms**	11/140 (8%)	17/174 (10%)	1.30	0.53	0.59	2.88	0.52
**Year ofs**	2011.41 [2000;2023]	2011.28 [2000;2023]	1.00	0.02	0.96	1.03	0.86

Multivariate model

	OR.	SE	p	95% CI Lower	95% CI Upper
**Age in years**	0.94	0.03	0.020	0.89	0.99
**pedNIHSS**	0.93	0.02	0.0036	0.89	0.98
**Hemiparesis**	0.80	0.24	0.46	0.44	1.45
**Headache**	0.62	0.21	0.16	0.32	1.20
**Speech problems**	0.94	0.27	0.84	0.53	1.66
**Facial palsy**	0.33	0.09	0.000096	0.19	0.57
**Year of stroke**	0.99	0.02	0.47	0.95	1.03

Above: univariate, below: multivariate logistic regression analyses for the whole cohort and the thrombectomy window. "n.p." indicates that regression was not possible due to insufficient case numbers. Swiss-SEP = Swiss Socioeconomic Position Index; PedNIHSS = Pediatric National Institutes of Health Stroke Scale; LoC = Level of Consciousness. Statistically significant results (p < 0.05) are shown in bold.

Table S11: Time from onset to diagnosis (TOD) – out-of-hospital AIS thrombectomy-window

	Descriptive		Logistic regression				
	**<24h Cont.: mean [min;max] Categ.: ratios(%)**	**>24h Cont.: mean [min;max] Categ.: ratios(%)**	**OR**	**SE**	**95% CI-Lower**	**95% CI-Upper**	**p**
**Age in years**	8.0 [0.32;15.95]	6.48 [0.18;15.99]	0.94	0.03	0.89	0.99	**0.019**
**Wakeup-AIS**	28/120 (23%)	14/111 (13%)	0.62	0.23	0.30	1.28	0.20
**Manifest. on weekend**	34/120 (28%)	32/111 (29%)	1.02	0.30	0.58	1.81	0.93
**≥1 transfer**	46/120 (38%)	54/111 (49%)	1.87	0.54	1.06	3.31	**0.031**
**Presentation**	36/120 (30%)	37/111 (33%)	0.59	0.11	0.40	0.86	**0.0060**
**Distance to next stroke center**	0/120 (0%)	0/111 (0%)	1.00	0.01	0.98	1.01	0.60
**Swiss-SEP**	61.81 [35.13;85.25]	61.01 [37.53;88.62]	0.99	0.01	0.97	1.02	0.57
**First image MRI**	56/120 (47%)	51/111 (46%)	0.96	0.25	0.57	1.61	0.87
**pedNIHSS**	7.94 [0;40]	5.39 [0;27]	0.91	0.03	0.86	0.96	**0.0012**
**Hemiparesis**	88/120 (73%)	70/111 (63%)	0.62	0.18	0.35	1.08	0.09
**Headache**	35/120 (29%)	27/111 (24%)	0.76	0.23	0.42	1.37	0.36
**Vertigo**	7/120 (6%)	16/111 (14%)	2.67	1.27	1.06	6.78	**0.038**
**Visual problems**	8/120 (7%)	9/111 (8%)	1.24	0.62	0.46	3.33	0.67
**Speech problems**	50/120 (42%)	40/111 (36%)	0.79	0.21	0.46	1.34	0.38
**Facial palsy**	61/120 (51%)	38/111 (34%)	0.50	0.14	0.29	0.85	**0.011**
**Balance problems**	48/120 (40%)	43/111 (39%)	0.95	0.26	0.56	1.61	0.85
**Decreased LoC**	43/120 (36%)	26/111 (23%)	0.55	0.16	0.31	0.97	**0.041**
**Seizures**	16/120 (13%)	18/111 (16%)	1.24	0.46	0.60	2.56	0.57
**Only non-specific symptoms**	1/120 (1%)	3/111 (3%)	3.31	3.85	0.34	32.29	0.30
**Only B&E-symptoms**	11/120 (9%)	15/111 (14%)	1.55	0.65	0.68	3.54	0.30
**Year of Stroke**	2011.26 [2000;2023]	2010.35 [2000;2023]	0.98	0.02	0.94	1.02	0.28

Multivariate model

	**OR.**	**SE**	**p**	**95% CI Lower**	**95% CI Upper**
**Age in years**	0.92	0.03	0.013	0.86	0.98
**≥1 transfer**	1.25	0.61	0.64	0.48	3.25
**Presentation**	0.65	0.20	0.17	0.35	1.20
**pedNIHSS**	0.91	0.03	0.010	0.85	0.98
**Vertigo**	3.87	2.38	0.028	1.16	12.92
**Facial asymmetry**	0.54	0.18	0.065	0.28	1.04
**Decreased LoC**	1.03	0.41	0.95	0.47	2.25
**Year of stroke**	0.97	0.03	0.32	0.92	1.03

Above: univariate, below: multivariate logistic regression analyses for out-of-hospital AIS and the thrombectomy window. "n.p." indicates that regression was not possible due to insufficient case numbers. Swiss-SEP = Swiss Socioeconomic Position Index; PedNIHSS = Pediatric National Institutes of Health Stroke Scale; LoC = Level of Consciousness. Statistically significant results (p < 0.05) are shown in bold.

Table S12: Time from onset to diagnosis (TOD) – In-hospital AIS thrombectomy-window

	Descriptive		Logistic regression				
	<24h Cont.: mean [min;max] Categ.: ratios(%)	>24h Cont.: mean [min;max] Categ.: ratios(%)	OR	SE	95% CI-Lower	95% CI-Upper	p
**Age in years**	5.97 [0.22;15.91]	4.36 [0.11;15.9]	0.94	0.05	0.86	1.04	0.23
**Manifest. on weekend**	5/20 (25%)	7/63 (11%)	0.37	0.25	0.10	1.35	0.13
**Swiss-SEP**	64.08 [31.82;87.52]	62.08 [28.49;83.94]	0.99	0.02	0.95	1.03	0.55
**First image MRI**	10/20 (50%)	30/63 (48%)	0.91	0.47	0.33	2.49	0.85
**pedNIHSS**	8.38 [1;23]	5.57 [0;25]	0.93	0.04	0.85	1.02	0.11
**Hemiparesis**	15/20 (75%)	35/63 (56%)	0.41	0.25	0.12	1.38	0.15
**Headache**	2/20 (10%)	2/63 (3%)	0.31	0.32	0.04	2.32	0.25
**Vertigo**	0/20 (0%)	2/63 (3%)	1.00				
**Visual problems**	2/20 (10%)	0/63 (0%)	1.00				
**Speech problems**	5/20 (25%)	4/63 (6%)	0.21	0.15	0.05	0.88	**0.032**
**Facial palsy**	9/20 (45%)	4/63 (6%)	0.08	0.06	0.02	0.32	**0.00031**
**Decreased LoC**	1/20 (5%)	34/63 (54%)	26.71	29.16	3.14	226.97	**0.0026**
**Seizures**	7/20 (35%)	25/63 (40%)	1.29	0.69	0.45	3.69	0.64
**Only non-specific symptoms**	0/20 (0%)	1/63 (2%)	1.00				
**Only B&E-symptoms**	0/20 (0%)	2/63 (3%)	1.00				
**Year of stroke**	2012.3 [2000;2022]	2012.9 [2000;2023]	1.01	0.04	0.94	1.09	0.72

Multivariate model

	OR.	SE	p	95% CI Lower	95% CI Upper
**Speech problems**	0.23	0.29	0.24	0.02	2.66
**Facial asymmetry**	0.78	0.95	0.84	0.07	8.38
**Decreased LoC**	22.99	26.04	0.0056	2.50	211.66
**Year of stroke**	1.00	0.07	0.99	0.88	1.14

Above: univariate, below: multivariate logistic regression analyses for in-hospital AIS and the thrombectomy window. "n.p." indicates that regression was not possible due to insufficient case numbers. Swiss-SEP = Swiss Socioeconomic Position Index; PedNIHSS = Pediatric National Institutes of Health Stroke Scale; LoC = Level of Consciousness. Statistically significant results (p < 0.05) are shown in bold.
